# Lipids, pH, and Their Interaction Affect the Inhibitory Effects of Carvacrol against *Salmonella* Typhimurium PT4 and *Escherichia coli* O157:H7

**DOI:** 10.3389/fmicb.2017.02701

**Published:** 2018-01-15

**Authors:** Rhayane I. Carvalho, Andrea S. de Jesus Medeiros, Maísa Chaves, Evandro L. de Souza, Marciane Magnani

**Affiliations:** ^1^Laboratório de Processos Microbianos em Alimentos, Departamento de Engenharia de Alimentos, Universidade Federal da Paraíba, Paraíba, Brazil; ^2^Laboratório de Microbiologia de Alimentos, Departamento de Nutrição, Centro de Ciências da Saúde, Universidade Federal da Paraíba, Paraíba, Brazil

**Keywords:** *Salmonella*, pathogenic *Escherichia coli*, terpenes, antibacterial effect, carvacrol

## Abstract

Although carvacrol (CAR) is considered an alternative antimicrobial for use in food, few is known about the influence of food-related parameters on its inhibitory effects against pathogens. This study assessed the influence of different amounts of proteins, using beef extract (BE) as a protein-rich source, lipids (LIP), using sunflower oil as a LIP-rich source, and pH values or their interaction on the inhibitory effects of CAR against *Salmonella* Typhimurium PT4 (ST) and *Escherichia coli* O157:H7 (EC). The specific maximum growth rate (*μmax*) and lag phase duration (λ) of the test pathogens when exposed to CAR in media with different amounts of BE (4, 6, and 8 g/100 mL), LIP (3.75, 5, and 6.25 mL/100 mL), and pH values (5, 5.5, and 6) were determined. The viable counts of the tested pathogens in media that promoted the highest and lowest *μmax* in the presence of CAR were monitored during 24 h. The lowest *μmax* of ST and EC exposed to 2.4 μL/mL (-1.29 and -0.82 log CFU/mL/h, respectively) or 4.8 μL/mL CAR (-1.44 and -2.17 log CFU/mL/h, respectively) were observed in media with the highest LIP amount (6.25 mL/100 mL) and pH value (pH 6). For both SE and EC, the longest λ (> 2 h) was verified in media where these pathogens showed the lowest *μmax*. These data indicate that the concomitant increase in LIP amounts and pH values affected positively the CAR inhibitory effects against the target pathogens. CAR (2.4 or 4.8 μL/mL) failed to inhibit the increase in ST and EC counts in media where the highest *μmax* values were previously observed. On the contrary, CAR inhibited the increase of ST counts (final counts 5 log CFU/mL) and decreased the EC counts (final counts 3.5 log CFU/mL) in media where the lowest *μmax* values were observed. These results show that the inhibitory effects of CAR on ST and EC in food matrices could be affected as a function of the interaction of LIP amounts and pH values.

## Introduction

Antimicrobial preservatives have been largely used to ensure food safety and reduce the risk to consumers (Tiwari et al., 2009; Lucera et al., 2012). Increasing consumer awareness and concern regarding the use of synthetic preservatives in foods has increased demand for foods with low amounts or free of synthetic preservatives (Lucera et al., 2012; Souza et al., 2016). In this context, efforts have been conducted to study natural substances capable of preventing bacterial growth and survival in foods (Hintz et al., 2015; de Souza et al., 2016).

Carvacrol [(2-methyl-5-(1-methyilethyl) fenol; CAR] is a plant-derived phenolic monoterpene well known as the majority constituent of essential oils (EOs) from *Origanum vulgare* L. (Rodrigues et al., 2017). CAR is also commonly identified as the majority constituent in EOs from *O. scabrum* L. (Aligiannis et al., 2001) and *Thymus vulgaris* L. (Sacchetti et al., 2005). Studies have attributed to CAR the bioactivities of these EOs, particularly their antibacterial effects against foodborne pathogens (Aligiannis et al., 2001; Perricone et al., 2015). The *in vitro* antibacterial properties of CAR have been demonstrated in different food matrices, such as vegetables (Oliveira et al., 2015), fruit juices (Ait-Ouazzou et al., 2013), peanut paste (Chen et al., 2015), cheese (Honório et al., 2015), and meat (Luz et al., 2014).

Carvacrol is Generally Recognized as Safe (GRAS) by the Food and Drug Administration in doses typically used as food flavoring and preservative ingredient (Regulation number of the United States Code of Federal Regulations 172.515) (Food and Drug Administration [FDA], 2013; Suntres et al., 2015). The available literature demonstrates that CAR is capable of inhibiting foodborne pathogens in very low doses compared to other EOs’ individual constituents (Honório et al., 2015; de Souza et al., 2016). Furthermore, CAR does not induce direct-tolerance and cross-tolerance to traditional antimicrobials used in foods (e.g., acids or salts) or heat treatment in pathogenic bacteria (Luz et al., 2013, 2014).

The food matrix composition may affect the efficacy of antimicrobials because the high availability of nutrients typically favors the fast recovery of injured bacterial cells (Burt, 2004; de Souza et al., 2016). Additionally, the structural organization of the components in food matrices may also limit the contact of antimicrobials with bacterial cells (Burt, 2004; Carvalho et al., 2015). Consequently, higher doses of antimicrobials could be required in food matrices to obtain the same antimicrobial effect as observed in *in vitro* assays (Perricone et al., 2015; Pesavento et al., 2015; de Souza et al., 2016). Carbohydrates typically do not affect the antimicrobial effects of EOs, while the pH and protein could affect their inhibitory effects against foodborne pathogens (Burt, 2004; Calo et al., 2015). However, there is a lack of studies assessing the effects of the interaction of pH with lipids or proteins on the inhibitory effects of EOs or their individual constituents.

Amongst the pathogens frequently involved in food-related outbreak diseases, *Salmonella* Typhimurium phage-type (PT) 4 and *Escherichia coli* O157H:7 receive attention because they have been most associated cause of outbreak-related hospitalizations in the United States (Centers for Diseases Control and Prevention [CDC], 2017). An important feature of these bacteria is the capability of growing in a wide variety of foods (e.g., fat-rich and protein-rich foods), as well as in acidic beverages (Espina et al., 2013; Melo et al., 2017; Moon et al., 2017).

This study evaluated the influence of different amounts of proteins, lipids, and pH values on the inhibitory effects of CAR on *S.* Typhimurium PT4 and *E. coli* O157:H7. The minimum inhibitory concentration (MIC) of CAR and the specific maximum growth rate and lag phase duration of the test pathogens when exposed to CAR in media with different amounts of beef extract (BE; as a protein rich-source), sunflower oil (as a lipid-rich source), and pH values were determined.

## Materials and Methods

### CAR

Carvacrol (batch 0656-810; density at 20°C: 0.976; refractive index at 20°C: 1.522) was purchased from Sigma–Aldrich (Sigma–Aldrich, St. Louis, MO, United States). CAR dispersions were prepared in brain heart infusion broth (BHIB; Himedia, India) in a concentration range of 40–0.312 μL/mL using Tween 80 (1 mL/100 mL; Sigma–Aldrich, United States) as an emulsifier (Rodrigues et al., 2017). At the highest assayed concentration (1 mL/100 mL), Tween 80 presented no inhibitory effect against the tested bacterial strains.

### Test Strains and Growth Conditions

*Salmonella enterica* serovar Typhimurium phage-type (PT) 4 (*S.* Typhimurium PT4) isolated from chicken meat involved in outbreak occurred in the South of Brazil (Kottwitz et al., 2011) and *E. coli* O157:H7 isolated from food sample (kindly provided by Prof. Anderson de Souza Sant’Ana, Quantitative Food Microbiology Laboratory, Food Engineering Faculty, São Paulo, Brazil) were used as test strains. Stocks were maintained in cryovials at -20°C in BHIB containing glycerol (20 mL/100 mL). Working cultures were maintained in brain heart infusion agar (BHIA, Himedia, India) at 4 ± 0.5°C, and transferred to a new medium weekly.

All experiments were performed following the Standard Microbiological Practices required for Biosafety Level 2 (Centers for Diseases Control and Prevention [CDC], 2015). The inoculum of each strain was obtained after preparing suspensions in sterile saline solution (NaCl 0.85 g/100 mL) from cultures grown in BHIB at 37°C for 18–20 h (stationary growth phase), harvested through centrifugation (4500 × *g*, 15 min, 4°C), washed twice, and re-suspended in sterile saline solution (NaCl 0.85 g/100 mL) to obtain standard cell suspensions. Optical density of 0.08 and 0.09 at 625 nm provided viable cell counts of approximately 6 log CFU/mL for *S.* Typhimurium PT4 and *E. coli* O157H:7, respectively (Melo et al., 2017).

### Preparation of Cultivation Media

The inhibitory effects of CAR on the test strains were evaluated in media prepared with different amounts of proteins (PTN), lipids (LIP), and pH values. The different media were used as models with the purpose of simulating possible environmental conditions found by microorganisms in a variety of food matrices with low-acidic to neutral pH (5, 5.5, and 6), low to medium amounts of LIP (3.75, 5, and 6.25 mL/100 mL), or distinct PTN contents (4, 6, and 8 g/100 mL of BE).

The assays followed a full factorial design 2^3^ as presented in **Table 1**, comprising 11 assays randomly performed. BE (75 g PTN/100g, Sigma–Aldrich, St. Louis, MO, United States) and sunflower oil (99 mL LIP/100 mL, Salada, Brazil) were used as a PTN- and LIP-rich source, respectively. To obtain the different media, BHIB was firstly added of BE at the desired amount, and sterilized in autoclave (121°C, 1.1 atm, 15 min). Subsequently, the corresponding amounts of sunflower oil previously sterilized (121°C, 1.1 atm, 15 min) were incorporated into the BHIB-BE media, vortexed for 15 s, and the pH value adjusted using HCl 1 mol/L (Gutierrez et al., 2009). The media were prepared in the same day of the experiments.

**Table 1:** Codified and absolute values of beef extract (BE), lipids (LIP), and pH used in cultivation media for assays with carvacrol (CAR) following a full factorial design (2^3^), and the respective minimal inhibitory concentration (MIC) of CAR against *S.* Typhimurium PT4 and *E. coli* O157H:7.

Media	Codified value *X*_1_ (BE)	Absolute value *X*_1_ (BE g/100 mL)	Codified value *X*_2_ (LIP)	Absolute value *X*_2_ (LIP mL/100 mL)	Codified value *X*_3_ (pH)	Absolute value *X*_3_ (pH)	MIC (μL/mL) *S.* Typhimurium PT4	MIC (μL/mL) *E. coli* O157H:7
1	-1.00	4.00	-1.00	3.75	-1.00	5.00	9.6	9.6
2	1.00	8.00	-1.00	3.75	-1.00	5.00	9.6	9.6
3	-1.00	4.00	1.00	6.25	-1.00	5.00	9.6	9.6
4	1.00	8.00	1.00	6.25	-1.00	5.00	9.6	9.6
5	-1.00	4.00	-1.00	3.75	1.00	6.00	9.6	9.6
6	1.00	8.00	-1.00	3.75	1.00	6.00	9.6	9.6
7	-1.00	4.00	1.00	6.25	1.00	6.00	9.6	4.8
8	1.00	8.00	1.00	6.25	1.00	6.00	9.6	4.8
9	0.00	6.00	0.00	5.00	0.00	5.50	9.6	9.6
10	0.00	6.00	0.00	5.00	0.00	5.50	9.6	9.6
11	0.00	6.00	0.00	5.00	0.00	5.50	9.6	9.6


### MIC Determination

The MIC values of CAR against the tested strains were determined using a previously described microdilution in broth procedure (Clinical and Laboratory Standards Institute [CLSI], 2012), with minor modifications related to the cultivation media and inoculum size. Initially, 50 μL of CAR emulsion in concentrations of 20–0.312 μL/mL in BHIB or 9.6–0.07 μL/mL in formulated media were dispensed into each well of a 96-well microplate. Subsequently, 50 μL of bacterial suspension (approximately 6 log CFU/mL) were added to each well. The microplate was loosely wrapped with cling wrap to prevent CAR volatilization. Each plate included controls without CAR. The systems were incubated at 37°C for 24 h. The MIC was defined as the lowest concentration of CAR capable of inhibiting the visible growth of the test strain (Carvalho et al., 2015).

### Assessing the Maximum Specific Growth Rate (*μmax*) and Lag Phase Duration (λ) of the Test Strains when Exposed to CAR

The growth of *S.* Typhimurium PT4 and *E. coli* O157:H7 was monitored in media containing 4.8 μL/mL (concentration corresponding to 1/2 MIC) or 2.4 μL/mL (concentration corresponding to 1/4 MIC) CAR in independent assays, using 96 well-microplates. Initially, 50 μL of the tested bacterial suspension (approximately 6 log CFU/mL) were added to each well containing 50 μL of the tested media. The bacterial growth was monitored by growth media turbidity in a microplate reader/incubator (EON, BioTek, United States) at 37°C each 2 h intervals during 24 h. Positive controls comprised the respective media inoculated with the test strain without CAR. Negative controls comprised the media containing CAR without the test strain. The *μmax* (log CFU/mL/h) and the λ (h) values were obtained from data of growth kinetics curves over time using the EON-Gen5 software (EON, BioTek, United States). For this, the microbial density read (turbidity of growth media) over the assessed time period in optical density at 625 nm (OD_625_) was log transformed into a logarithmic scale to obtain the logOD_625_/time-curve of the bacterial population after subtraction of the negative control (Gutierrez et al., 2008, 2009). Considering the logOD_625_/time-curve, the λ was the time of the transition to the exponential phase after the initial population had doubled. To obtain these parameters, EON-Gen5 calculated the value of the maximum slope (*μmax*) as follow: starting at the first time-point, the slope among the *n*-points of the time period selected for evaluation was evaluated. The operation was repeated starting at the second point, and repeated again, starting at the third point, and so on. Finally, all calculated slopes were compared to determine the maximum slope. The λ was the time interval between the line of maximum slope of the propagation phase and the absorbance baseline at time = 0 (EON-Gen5 software, EON, BioTek, United States).

### Assessing the Viable Counts of the Test Strains during Exposure to CAR in Selected Media

The viable counts of *S.* Typhimurium PT4 and *E. coli* O157:H7 were assessed during 24 h of incubation at 37°C in media that provided the highest and the lowest *μmax* values in turbidity assays (see section “Assessing the Maximum Specific Growth Rate (*μmax*) and Lag Phase Duration (λ) of the Test Strains when Exposed to CAR”). Viable counts of *S.* Typhimurium PT4 were assayed in media 6 and 8 containing 4.8 μL/mL CAR and media 3 and 7 containing 2.4 μL/mL CAR. Viable counts of *E. coli* O157:H7 were assayed in media 2 and 8 containing 4.8 μL/mL CAR and media 5 and 7 containing 2.4 μL/mL CAR (**Table 1**). Initially, 20 μL of each bacterial suspension (approximately 6 log CFU/mL) were inoculated into 3480 μL of the selected media containing CAR. The mixtures (final viable counts of approximately 5 log CFU/mL) were gently hand-shaken for 30 s and subsequently incubated at 37°C. At intervals of 0 (just after homogenization), 2, 4, 6, 8, 12, 14, 16, and 24 h of cultivation, a 100 μL-aliquot of each mixture was serially diluted in sterile saline solution, inoculated using a microdrop inoculation technique (Herigstad et al., 2001) on BHIA and incubated at 37°C for 24 h. Control media without CAR were similarly assayed. The results are expressed as log CFU/mL. The detection limit of the test was 2 log CFU/mL.

### Statistical Analysis and Reproducibility

All assays were performed in triplicate in three independent experiments and the results are expressed as an average of the obtained data. For MIC determination assays, the results are expressed as modal values because the MIC values did not vary in the independent experiments (Souza et al., 2016). For the *μmax*, λ, and viable counts assays, statistical analysis were performed to determine significant differences (*p* ≤ 0.05) using analysis of variance (ANOVA) followed by Tukey’s test or Student’s *t*-test. The results of *μmax* from turbidity assays were analyzed using response surface methodology (RSM) and the goodness-of-fit and statistical validity of the models generated by RSM were assessed by using one-factor ANOVA, as well as by providing the adjusted determination coefficient (*R*^2^adj) and the statistical significance of the regression models (*p*-value 0.05) (Bernardes et al., 2012). All analyses were performed using the Statistica software version 7.0 (StatSoft, Inc., Tulsa, OK, United States).

## Results

### MIC of CAR in Different Media

The MIC of CAR on *S.* Typhimurium PT4 was 9.6 μL/mL in all the different formulated media. The MIC of CAR on *E. coli* O157:H7 in different formulated media was 4.8 or 9.6 μL/mL (**Table 1**).

### *μmax* and λ of the Test Strains Exposed to CAR

The *μmax* of *S.* Typhimurium PT4 was higher (*p* ≤ 0.05) in media without CAR than in media containing 4.8 or 2.4 μL/mL CAR (**Table 2**). The lowest *μmax* of *S.* Typhimurium PT4 (-1.44 log CFU/mL/h) exposed to 4.8 μL/mL CAR was observed in medium with the highest amount of BE (8 g/100 mL) and LIP (6.25 mL/100 mL) at pH 6.0 (medium 8). *S.* Typhimurium PT4 (-1.29 CFU/mL/h) showed the lowest *μmax* when exposed to 2.4 μL/mL CAR in medium with 4 g/100 mL BE and 6.25 mL/100 mL LIP at pH 6.0 (medium 7). The highest *μmax* of *S.* Typhimurium PT4 (4.63 CFU/mL/h) exposed to 4.8 μL/mL CAR was observed in medium with 8 g/100 mL BE and 3.75 mL/100 mL LIP at pH 6.0 (medium 6). The highest *μmax* of *S.* Typhimurium PT4 (2.39 CFU/mL/h) exposed to 2.4 μL/mL CAR was observed in medium with 4 g/100 mL BE and 6.25 mL/100 mL LIP at pH 5.0 (medium 3) (**Table 2**).

**Table 2:** Maximum specific growth rate (*μmax*; log CFU/mL/h) and lag phase (λ; h) of *S.* Typhimurium PT4 grown in media containing different concentrations of carvacrol (CAR).

Media	Control *μmax* (log CFU/mL/h)	CAR 4.8 *μmax* (log CFU/mL/h)	CAR 4.8^∗^ λ (h)	CAR 2.4 *μmax* (log CFU/mL/h)	CAR 2.4^∗∗^ λ (h)
1	5.33 ± 0.20^Ac^	1.80 ± 0.15^Ba^	0.00 ± 0.00	2.39 ± 0.17^Be^	0.00 ± 0.00
2	4.50 ± 0.11^Ae^	1.82 ± 0.10^Ba^	0.00 ± 0.00	3.90 ± 0.28^Bf^	0.00 ± 0.00
3	5.01 ± 0.26^Aac^	2.39 ± 0.29^Bb^	0.00 ± 0.00	2.64 ± 0.23^Be^	0.00 ± 0.00
4	4.37 ± 0.07^Af^	1.28 ± 0.08^Bc^	0.00 ± 0.00	3.83 ± 0.21^Bf^	0.00 ± 0.00
5	4.95 ± 0.06^Aab^	1.79 ± 0.15^Ba^	0.00 ± 0.00	3.25 ± 0.15^Bg^	0.00 ± 0.00
6	5.12 ± 0.08^Aac^	1.80 ± 0.06^Ba^	0.00 ± 0.00	4.63 ± 0.20^Ba^	0.00 ± 0.00
7	3.00 ± 0.08^Ag^	-1.29 ± 0.36^Bd^	3.48 ± 0.03^a^	1.45 ± 0.06^Bc^	0.00 ± 0.00
8	2.09 ± 0.04^Ad^	-0.88 ± 0.03^Be^	2.30 ± 0.01^b^	-1.44 ± 0.07^Bb^	2.40 ± 0.00^a^
9	2.33 ± 0.02^Ad^	1.97 ± 0.02^Ba^	0.00 ± 0.00	1.94 ± 0.05^Bd^	0.00 ± 0.00
10	2.37 ± 0.10^Ad^	1.95 ± 0.05^Ba^	0.00 ± 0.00	1.91 ± 0.03^Bd^	0.00 ± 0.00
11	2.35 ± 0.02^Ad^	1.89 ± 0.01^Ba^	0.00 ± 0.00	1.91 ± 0.06^Bd^	0.00 ± 0.00


*Control: same formulation media without addition of CAR; CAR 4.8: media containing 4.8 μL/mL CAR; ^∗^ or ^∗∗^ all media assayed as control of CAR 4.8 or CAR 2.4 showed λ-value = 0; CAR 2.4: media containing 2.4 μL/mL CAR. ^A,B^Different superscript capital letters in the same raw denote differences (*p* ≤ 0.05) in maximum specific growth rate obtained for each bacteria based on Student’s *t*-test; ^a-g^Different superscript small letters in the same column denote differences (*p* ≤ 0.05) in maximum specific growth rate and lag phase obtained for each bacteria based on Tukey’s test.*

The λ of *S.* Typhimurium PT4 varied from 0 to 3.48 h when the strain was exposed to 4.8 μL/mL CAR and from 0 to 2.4 h when this strain was exposed to 2.4 μL/mL CAR. *S.* Typhimurium PT4 showed the longest λ when exposed to 2.4 or 4.8 μL/mL CAR in medium with the same amount of LIP (6.25 mL/100 mL) at pH (6.0) but containing 4 g/100 mL (medium 7) and 8 g/100 mL (media 8) BE, respectively (**Table 2**).

Similarly to observed for *S.* Typhimurium PT4, the *μmax* of *E. coli* O157:H7 was always higher (*p* ≤ 0.05) in media without CAR than in media containing 4.8 or 2.4 μL/mL CAR (**Table 3**). The lowest *μmax* of *E. coli* O157:H7 (-2.17 CFU/mL/h) exposed to 4.8 μL/mL CAR was observed in medium with the highest amount of BE (8 g/100 mL) and LIP (6.25 mL/100 mL) at pH 6.0 (medium 8). *E. coli* O157:H7 (-1.02 CFU/mL/h) showed the lowest *μmax* when exposed to 2.4 μL/mL CAR in medium with 4 g/100 mL BE and 6.25 mL/100 mL LIP at pH 6.0 (medium 7). The highest *μmax* of *E. coli* O157:H7 (2.85 CFU/mL/h) exposed to 4.8 μL/mL CAR was observed in medium with 8 g/100 mL BE and 3.75 mL/100 mL LIP at pH 5.0 (medium 2). The highest *μmax* of *E. coli* O157:H7 exposed to 2.4 μL/mL CAR was observed in medium with 4 g/100 mL BE and 3.75 mL/100 mL LIP at pH 6.0 (medium 5) (**Table 3**).

**Table 3:** Maximum specific growth rate (*μmax*; log CFU/mL/h) and lag phase (λ; h) of *E. coli* O157H:7 grown in media containing different concentrations of carvacrol (CAR).

Media	Control *μmax* (log CFU/mL/h)	CAR 4.8^∗^ *μmax* (log CFU/mL/h)	CAR 4.8^∗^ λ (h)	CAR 2.4 *μmax* (log CFU/mL/h)	CAR 2.4^∗∗^ λ (h)
1	4.43 ± 0.08^Ae^	0.73 ± 0.14^Be^	0.00 ± 0.00	1.95 ± 0.16^Be^	0.00 ± 0.00
2	3.90 ± 0.04^Af^	2.85 ± 0.08^Bf^	0.00 ± 0.00	2.33 ± 0.13^Bc^	0.00 ± 0.00
3	2.20 ± 0.04^Ab^	1.86 ± 0.01^Babcd^	0.00 ± 0.00	2.50 ± 0.19^Bc^	0.00 ± 0.00
4	1.93 ± 0.01^Acd^	1.53 ± 0.14^Bab^	0.00 ± 0.00	1.60 ± 0.08^Bd^	0.00 ± 0.00
5	2.44 ± 0.02^Aab^	1.90 ± 0.21^Bcd^	0.00 ± 0.00	3.45 ± 0.11^Ba^	0.00 ± 0.00
6	1.72 ± 0.01^Ad^	1.25 ± 0.16^Bg^	0.00 ± 0.00	2.33 ± 0.19^Bc^	0.00 ± 0.00
7	1.69 ± 0.08^Ad^	-1.39 ± 0.17^Bh^	4.30 ± 0.03^a^	-1.02 ± 0.11^Bb^	1.77 ± 0.01
8	1.61 ± 0.07^Ad^	-2.17 ± 0.29^Bi^	4.00 ± 0.03^b^	-0.82 ± 0.21^Bb^	2.32 ± 0.01^a^
9	2.52 ± 0.12^Aa^	1.66 ± 0.01^Babd^	0.00 ± 0.00	1.98 ± 0.01^Bde^	1.02 ± 0.01^c^
10	2.25 ± 0.21^Aac^	1.70 ± 0.01^Babd^	0.00 ± 0.00	1.79 ± 0.01^Bde^	1.80 ± 0.00^c^
11	2.45 ± 0.25^Aab^	1.68 ± 0.03^Babd^	0.00 ± 0.00	1.86 ± 0.05^Bde^	1.78 ± 0.00^c^


*Control: same formulation media without addition of CAR; CAR 4.8: media containing 4.8 μL/mL CAR; ^∗^ or ^∗∗^ all media assayed as control of CAR 4.8 or CAR 2.4 showed λ-value = 0; CAR 2.4: media containing 2.4 μL/mL CAR. ^A,B^Different superscript capital letters in the same raw denote differences (*p* ≤ 0.05) in maximum specific growth rate obtained for each bacteria based on Student’s *t*-test; ^a-i^Different superscript small letters in the same column denote differences (*p* ≤ 0.05) in maximum specific growth rate and lag phase obtained for each bacteria based on Tukey’s test.*

The λ of *E. coli* O157:H7 varied from 0 to 4.30 h when the strain was exposed to 4.8 μL/mL CAR, and from 0 to 2.32 h when the strain was exposed to 2.4 μL/mL CAR (**Table 3**).

The Eqs 1 and 2 explain the effects of LIP and/or pH as well as of their interaction on the *μmax* of *S.* Typhimurium PT4 obtained in assays using 4.8 μL/mL (*S*T4.8) and 2.4 μL/mL (*S*T2.4) CAR, respectively. Similarly, the Eqs 3 and 4 explain the effects of LIP and pH as well as of their interaction on the *μmax* of *E. coli* O157:H7 in assays using 4.8 μL/mL (*Ec*4.8) and 2.4 μL/mL (*Ec*2.4) CAR, respectively. All models included only significant variables (*p* < 0.05) and their determination coefficient (*R*^2^adj) >0.85 showed a good fit to the experimental data.

$$Y_{\mu \,\max}(ST4.8)=1.32-1.42\,\;X_{2}-1.46\,\;X_{3}-1.45\,\;X_{2}\cdot X_{3}\left(R^{2}=0.88\right)$$

$$Y_{\mu \,\max}(ST2.4)=2.4-1.92\,\;X_{2}-2.01\,\;X_{2}\cdot X_{3}\left(R^{2}=0.89\right)$$

$$Y_{\mu \,\max}(Ec4.8)=1.05-1.72\,\;X_{2}-1.84\,\;X_{3}-1.63\,\;X_{2}\cdot X_{3}\left(R^{2}=0.90\right)$$

$$Y_{\mu \,\max}(Ec2.4)=1.63-1.95\,\;X_{2}-1.11\,\;X_{3}-1.86\,\;X_{2}\cdot X_{3}\left(R^{2}=0.94\right)$$

where *X*_2_ = LIP, *X*_3_ = pH, and *Y_μ_*_max_ = specific maximum growth rate.

**FIGURE 1:**
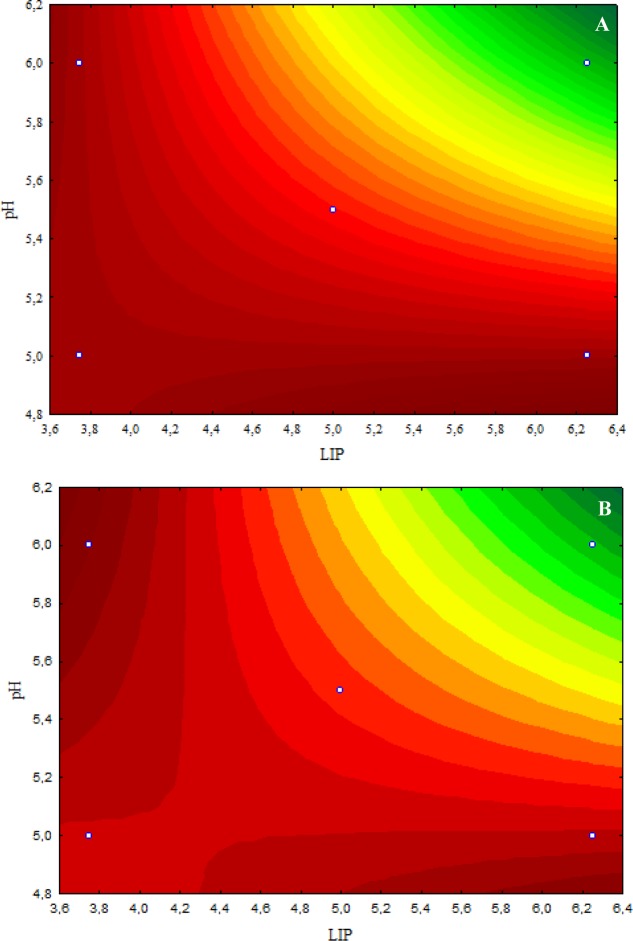
**(A)** Curves of surface response of *μmax* obtained from cultivation of *S.* Typhimurium PT4 in media with different amounts of LIP and pH values and 4.8 μL/mL carvacrol (CAR). **(B)** Curves of surface response of *μmax* obtained from cultivation of *S.* Typhimurium PT4 in media with different amounts of LIP and pH values and 2.4 μL/mL CAR.

According to the response surface contours, the inhibitory effects caused by 4.8 μL/mL CAR on *S.* Typhimurium decreased when the LIP amount decreased (LIP effect) or when the LIP amount decreased and the pH increased (LIP–pH interaction effect) (**Figure 1A**). The inhibitory effect caused by 2.4 μL/mL CAR on *S.* Typhimurium was affected by LIP amount (LIP effect), pH values (pH effect), or their interaction (LIP–pH interaction effect); the lowest inhibitory effect of CAR was observed in medium prepared with the highest LIP amount and the lowest pH value (**Figure 1B**).

**FIGURE 2:**
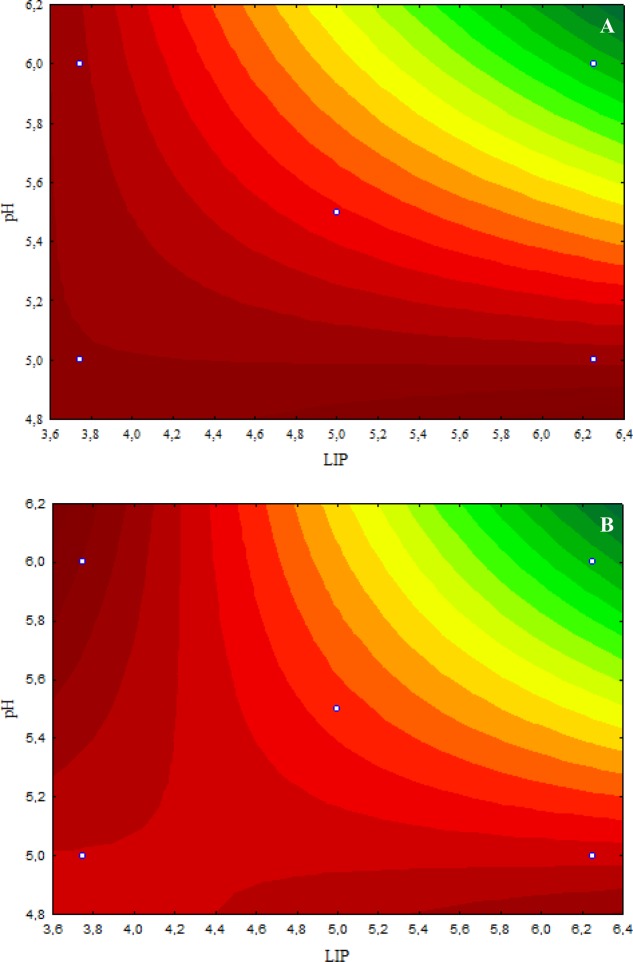
**(A)** Curves of surface response of *μmax* obtained from cultivation of *E. coli* O157H:7 in media with different amounts of LIP and pH values and 4.8 μL/mL carvacrol (CAR). **(B)** Curves of surface response of *μmax* obtained from cultivation of *E. coli* O157H:7 in media with different amounts of LIP and pH values and 2.4 μL/mL CAR.

The inhibitory effect caused by 4.8 μL/mL CAR on *E. coli* O157:H7 was decreased when the LIP amount decreased (LIP effect), pH decreased (pH effect), or when the LIP amount decreased and pH increased (LIP–pH interaction effect) (**Figure 2A**). The lowest inhibitory effects of CAR were observed in media prepared with the lowest LIP amount at the lowest pH value (**Figure 2A**). Similar effects of LIP, pH, or their interaction were observed for the inhibitory effects caused by 2.4 μL/mL CAR on *E. coli* O157:H7 (**Figure 2B**).

The BE amount added in the cultivation media did not influence the inhibitory effects of CAR against the test pathogens under the experimental conditions assayed. The data were experimentally validated in three independent experiments performed under the optimized conditions according to the analysis of the RSM.

### Effects of CAR on Bacterial Counts in Selected Media

**FIGURE 3:**
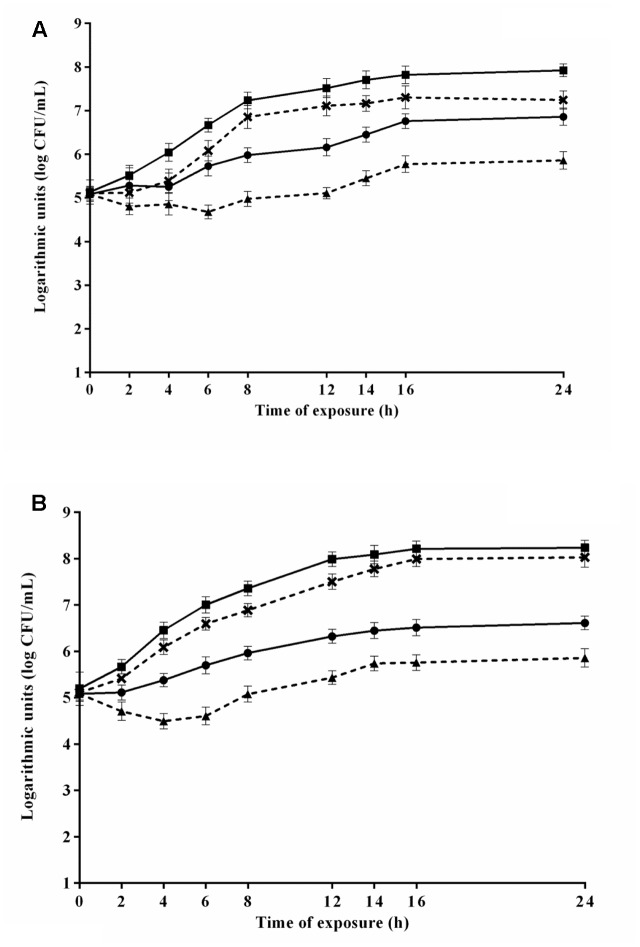
Viable cell counts of *S.* Typhimurium PT4 in media at 37°C during exposure to **(A)** 4.8 μL/mL or **(B)** 2.4 μL/mL carvacrol (CAR). (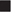) Counts in media corresponding to the highest *μmax* (6 or 3) without addition of CAR; (×) counts in media corresponding to the highest *μmax* (6 or 3); (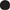) counts of media corresponding to the lowest *μmax* without CAR (8 or 7); (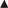) counts in media corresponding to the lowest *μmax* (8 or 7). Detection limit of the test: 2.0 log CFU/mL. The error bars represent the standard deviation.

The viable counts of *S.* Typhimurium PT4 during 24 h-exposure to 4.8 μL/mL CAR in media 6 and 8 that provided the highest and lowest *μmax*, respectively, are shown in **Figure 3A**. *S.* Typhimurium PT4 exhibited lower counts (*p* ≤ 0.05) in media containing CAR compared to media without CAR. An increase (*p* ≤ 0.05) of approximately 2 log units in counts of *S.* Typhimurium PT4 was observed in the earlier 8 h of exposure to CAR in medium 6, with no increase (*p* > 0.05) in counts in the further monitored exposure time intervals. No changes were observed in counts of *S.* Typhimurium PT4 during the 24 h of exposure to 4.8 μL/mL CAR in medium 8 (selected because provided the lowest *μmax*). An increase (*p* ≤ 0.05) near to 3 log units was observed in counts of *S.* Typhimurium PT4 in the earlier 16 h of exposure to 2.4 μL/mL CAR in medium 3 (selected because provided the highest *μmax*). Otherwise, no changes (*p* > 0.05) were observed in counts of *S.* Typhimurium PT4 during the 24 h of exposure to 2.4 μL/mL CAR in medium 7 (selected because provided the lowest *μmax*) (**Figure 3B**).

**FIGURE 4:**
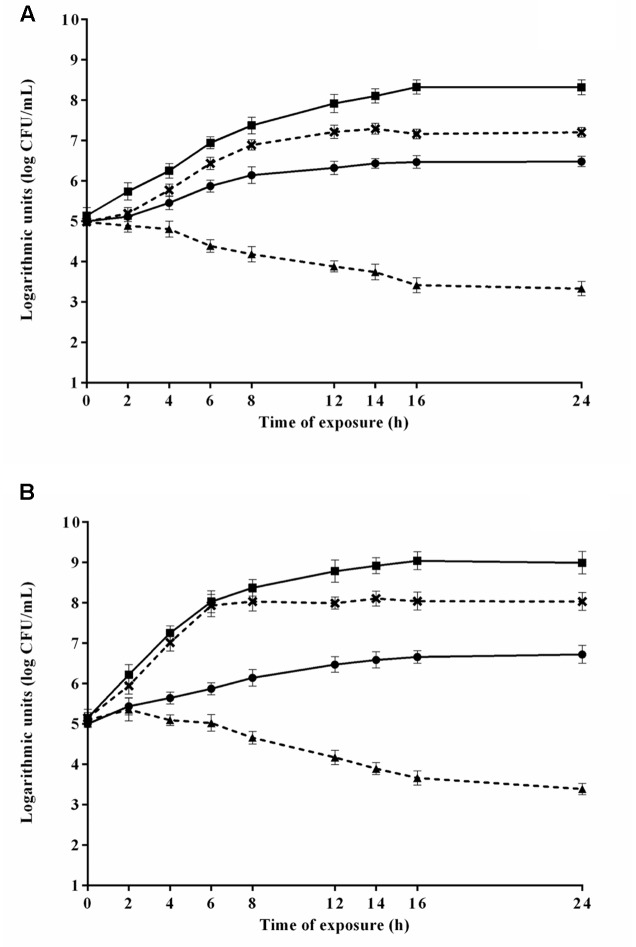
Viable cell counts of *E. coli* O157H:7 in media at 37°C during exposure to **(A)** 4.8 μL/mL or **(B)** 2.4 μL/mL carvacrol (CAR). (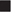) Counts in media corresponding to the highest *μmax* (3 or 2) without CAR; (×) counts in media corresponding to the highest *μmax* (3 or 2); (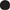) counts in media corresponding to the lowest *μmax* without CAR (7 or 8); (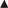) counts in media corresponding to the lowest *μmax* (7 or 8). Detection limit of the test: 2.0 log CFU/mL. The error bars represent the standard deviation.

The counts of *E. coli* O157H:7 during the 24 h exposure to 4.8 μL/mL CAR in media 2 and 8 (selected because provided the highest and lowest *μmax*, respectively) are shown in **Figure 4A**. *E. coli* O157H:7 exhibited lower counts (*p* ≤ 0.05) in media containing CAR compared to media without CAR. *E. coli* O157:H7 increased the counts (*p* ≤ 0.05) in approximately 2 log units in the earlier 8 h of exposure to CAR in medium 2, with no changes (*p* > 0.05) in counts in the further monitored exposure time intervals. A decrease (*p* ≤ 0.05) near to 1.5 log units in counts of *E. coli* O157:H7 was observed after 12 h of exposure to 4.8 μL/mL CAR in medium 8 (**Figure 4A**). An increase (*p* ≤ 0.05) near to 3 log units was observed in counts of *E. coli* O157H:7 in the earlier 6 h of exposure to 2.4 μL/mL CAR in medium 5 (selected because provided the highest *μmax*). Otherwise, a decrease (*p* ≤ 0.05) of approximately 1.5 log were observed in initial counts of *E. coli* O157H:7 exposed to 2.4 μL/mL CAR in medium 7 (selected because promoted the lowest *μmax*) over the monitored exposure time period (**Figure 4B**).

## Discussion

The highest inhibitory effects of CAR were observed in media with the highest LIP amounts and pH value tested despite of the BE or CAR amounts. The influence of LIP, pH, or their interaction on CAR antibacterial effects, according to the models of Eqs 1–4, explain the increased *μmax* of both tested pathogens during exposure to CAR in media with the lowest LIP amount assayed. Overall, the effects of LIP, pH, and their interaction explain the negative *μmax* observed for the test pathogens during exposure to CAR in media with the highest LIP amount assayed at pH 6.0.

The LIP–pH interaction probably caused the decreased inhibitory effects of CAR on *S.* Typhimurium PT4 and *E. coli* O157:H7 in acidic media, in opposite to reports of previous studies (Burt, 2004; Gutierrez et al., 2009; Negi, 2012). The negative impact of LIP on antimicrobial effects of phenolics has been associated with the decrease availability of the later in aqueous phase, which primarily binds to LIP and, consequently, do not target the bacterial cells that are mostly in aqueous phase of growth substrates (Smith-Palmer et al., 2001; Perricone et al., 2015). An earlier study reported decrease of CAR inhibitory effect on *Salmonella* Tennesse in high-LIP peanut paste (50% LIP). However, in the same study, CAR failed to inhibit *Salmonella* growth in low-LIP peanut (<5%) when the a_w_ increased as a result of the interaction of growth substrate intrinsic factors (Chen et al., 2015).

Probably, in the conditions tested in this study, the effects of the pH in media might limit the CAR–LIP affinity and, consequently, CAR maintained the efficacy to inhibit the test pathogens even in high-LIP amounts. The presence of high-LIP amounts in the media may promote changes on the extracellular environment, which could increase the interaction of CAR (as a prevalent hydrophobic molecule) with the bacterial cytoplasmic membrane (Ultee et al., 2002; Raei et al., 2017). Environmental characteristics that facilitate CAR to reach bacterial membranes have been considered important factors to enhance their antibacterial efficacy (Knowles and Roller, 2001).

Interestingly, a previous study reported decrease of the *μmax* of *L. monocytogenes* in media with 10 g/100 mL LIP and oregano EO (CAR-chemotype), suggesting that the added-LIP amount enhanced the antibacterial efficacy of the tested EO (Gutierrez et al., 2008); however, the effects of pH or a possible LIP–pH interaction were not considered.

The λ of *S.* Typhimurium PT4 and *E. coli* O157:H7 varied in media containing or not CAR (2.4 or 4.8 μL/mL). For both the tested pathogens, the longest λ was observed in media where the strains showed the lowest *μmax.* Probably, the stress caused by the exposure to CAR in these media delayed the bacterial cell metabolism, requiring a longer time for bacterial cell adaptation and further increase in bacterial cell population (Lee et al., 2015; Suntres et al., 2015).

Counts of *S.* Typhimurium PT4 increased during exposure to CAR in media that promoted the highest *μmax* of this pathogen, showing that CAR failed to inhibit the growth of *S.* Typhimurium PT4 over time. It is interesting to note that in these media the test strain did not require time for adaptation (λ = 0) even in the presence of CAR. Probably, the negative effects of LIP and pH on CAR inhibitory effects in a media with high availability of nutrients induced a fast and intense bacterial metabolic activity. Otherwise, in media where *S.* Typhimurium PT4 showed the lowest *μmax* and longer λ, CAR exhibited bacteriostatic effects delaying the bacterial growth over time.

Counts of *E. coli* O157H:7 increased in media where the pathogen showed the highest *μmax* and decreased in media where the lowest *μmax* was observed despite of the CAR amount. Thus, CAR exerted antibacterial effects even at sub-MICs in media where pH and LIP did not affect its efficacy. CAR uptake through the outer bacterial membrane is self-promoted because of the CAR ability to interact with the structure of the lipopolysaccharide barrier (Luz et al., 2014). This feature may facilitate the action of CAR even at sub-MICs because once into the cell, CAR can cause a disturbance in protein synthesis and functions (Sousa et al., 2015).

To the best of our knowledge, this is the first study that assessed the effects of the LIP–pH interaction on the inhibitory effects of CAR against *S.* Typhimurium PT4 and *E. coli* O157H:7. The results showed that the increase in LIP amounts and pH values enhances the inhibitory effects of CAR on *S.* Typhimurium PT4 and *E. coli* O157H:7, primarily as a result of the LIP-pH interaction. These findings indicate that the influence of LIP and pH on the antibacterial effects of CAR should be considered for the use of this compound as an antimicrobial preservative in food matrices to avoid failure on the expected microbial control.

## Author Contributions

MM and ELS conceived and designed the experiments. RIC, AJM, and MC performed the experiments. RIC, ELS, and MM analyzed the data and drafted the paper.

## Conflict of Interest Statement

The authors declare that the research was conducted in the absence of any commercial or financial relationships that could be construed as a potential conflict of interest.
